# Expansion of health facilities in Iraq a decade after the US-led invasion, 2003–2012

**DOI:** 10.1186/1752-1505-8-16

**Published:** 2014-09-11

**Authors:** Valeria Cetorelli, Nazar P Shabila

**Affiliations:** 1 Department of Social Policy, London School of Economics and Political Science, Houghton Street, WC2A 2AE London, UK; 2 Department of Community Medicine, College of Medicine, Hawler Medical University, Erbil, Iraq

**Keywords:** Iraq, Fragile and conflict affected states, Health system rehabilitation, Health facilities

## Abstract

**Background:**

In the last few decades, Iraq’s health care capacity has been severely undermined by the effects of different wars, international sanctions, sectarian violence and political instability. In the aftermath of the 2003 US-led invasion, the Ministry of Health has set plans to expand health service delivery, by reorienting the public sector towards primary health care and attributing a larger role to the private sector for hospital care. Quantitative assessments of the post-2003 health policy outcomes have remained scant. This paper addresses this gap focusing on a key outcome indicator that is the expansion of health facilities.

**Methods:**

The analysis is based on data on health facilities provided by the World Health Organisation and Iraq’s Ministry of Health. For each governorate, we calculated the change in the absolute number of facilities by type from early 2003 to the end of 2012. To account for population growth, we computed the change in the number of facilities per 100,000 population. We compared trends in the autonomous northern Kurdistan region, which has been relatively stable from 2003 onwards, and in the rest of Iraq (centre/south), where fragile institutions and persistent sectarian strife have posed major challenges to health system recovery.

**Results:**

The countrywide number of primary health care centres per 100,000 population rose from 5.5 in 2003 to 7.4 in 2012. The extent of improvement varied significantly within the country, with an average increase of 4.3 primary health care centres per 100,000 population in the Kurdistan region versus an average increase of only 1.4 in central/southern Iraq. The average number of public hospitals per 100,000 population rose from 1.3 to 1.5 in Kurdistan, whereas it remained at 0.6 in centre/south. The average number of private hospitals per 100,000 population rose from 0.2 to 0.6 in Kurdistan, whereas it declined from 0.3 to 0.2 in centre/south.

**Conclusions:**

The expansion of both public and private health facilities in the Kurdistan region appears encouraging, but still much should be done to reach the standards of neighbouring countries. The slow pace of improvement in the rest of Iraq is largely attributable to the dire security situation and should be a cause for major concern.

## Background

Health systems suffer a heavy toll in fragile and conflict affected states [1]. Iraq is an exemplifying case. Throughout the 1970s and 1980s, Iraq’s health system used to be one of the most advanced in the Middle East [2]. The system was highly centralised, hospital-oriented and fully government-subsided with revenues from the nationalised oil industry [3]. However, in the last few decades the country’s health care capacity has been severely undermined by the effects of different wars, international sanctions, sectarian violence and political instability.

Since the 1980–1988 Iran-Iraq War, resources were progressively diverted from the health sector [2]. During the 1990–1991 Gulf War and the following 13 years of embargo and economic sanctions, public health budget was cut by 90% and buildings and equipment fell into disrepair [2]. At the time of the 2003 US-led invasion, serious damages occurred from widespread looting and destruction of facilities [4]. The violence-induced exodus of thousands of doctors and nurses in the subsequent years further weakened the health system [5].

The urgency of health care rehabilitation was clear in the aftermath of the invasion. After 2003, Iraq’s Ministry of Health has set plans to expand health service delivery, moving towards a decentralised primary health care model [6]. National development plans have also called for the emergence of a private sector, which may potentially contribute to enhance the provision of secondary and tertiary care [7]. The separate Ministry of Health of the autonomous Iraqi Kurdistan region has shared a similar approach, namely a reorientation of the public sector towards primary health care and a larger role to the private sector for hospital care [8].

The shortcomings of the post-2003 health policy framework have been discussed extensively, in particular its lack of specificity and commitment to clear long-term objectives [9,10]. Nevertheless, quantitative assessments of the policy outcomes have remained scant. This paper addresses this gap focusing on a key outcome indicator that is the expansion in number, type and location of health facilities per population. The study intends to contribute to the growing body of academic and policy literature on post-conflict health system recovery.

Strengthening health infrastructure is deemed a critical component for health system recovery in Iraq as elsewhere [11]. Virtually all health strategies in countries emerging from conflict include plans for an adequate network of equitably distributed health facilities to meet the population’s health needs [12-16]. Studies have shown that successful infrastructure programmes, such as the expansion of health facilities in underserved areas, increase access to services and may also foster the process of peace building and state legitimacy [17-20].

However, this is an arduous and complex undertaking [21]. Previous research has stressed the importance of inclusive political settlements to bring the stability required to allow a successful implementation of any reconstruction and development plans [22,23]. Such stability has clearly lacked in post-2003 Iraq, characterised by fragile institutions and persistent sectarian strife [24,25]. The situation has been different in the autonomous Kurdistan region. Unlike the rest of the country, this region has not suffered from generalised violence and political uncertainty, and this has guaranteed more favourable conditions for development [9].

These differences in political context within the country make Iraq a useful case study to assess variation in health policy outcomes. The following analysis compares changes in the number of health facilities per population in the autonomous Kurdistan region, which has been relatively stable from 2003 onwards, and in the rest of Iraq, where persistent insecurity has posed major challenges to health system recovery. The focus is on the expansion of primary health care centres (PHCCs), public hospitals and private hospitals a decade after the US-led invasion. We discuss the insights gained from such comparison and suggest policy implications for the coming years.

### Iraq’s health system

The organisational structure of the Iraqi health system dates back to 1970s and consists of two main levels: the Ministry of Health as a central planning level, and the Directorates of Health as a local administrative level in each governorate [3]. After the Gulf War, the three northern Kurdish governorates of Dohouk, Erbil and Al-Sulaimaniya became a de facto autonomous region under UN auspices, and a separate Ministry of Health was established for the Kurdistan regional government with much the same structure [26].

In the public sector, health services are provided through a network of PHCCs and public hospitals at very low charges. The PHCCs provide preventive and basic curative services. The main centres are located in urban areas and are typically administered by doctors, while smaller centres are located in rural areas and are generally staffed with medical auxiliaries only [27]. Recent surveys have highlighted significant impediments to delivering adequate services in the PHCCs, including poor organisation and shortage of manpower and medications [28,29]. Despite numerous problems, the PHCCs are recognised as very important sources of health care provision, particularly for the poor [30].

For secondary and tertiary care, patients are referred from PHCCs to hospitals. However, it is estimated that only about 40% of Iraqis have access to referral services due to the inadequate number and uneven distribution of public hospitals [31]. Secondary and tertiary care are also provided by small private hospitals. Since there are no health insurance schemes in Iraq, private health care is met out-of-pocket and is well beyond the reach of many Iraqis [21]. Moreover, although private hospitals are licensed by the Ministry of Health, they are still largely outside the national health supervision system [32].

## Methods

This study is based on data on health facilities provided by the World Health Organisation (WHO) and Iraq’s Ministry of Health for the years 2003 and 2012 respectively. In early 2003, the WHO published a detailed record of all functioning health facilities for each Iraqi governorate by type. The inventory and categorisation of facilities was carried out by the WHO staff a few months before the US-led invasion and was part of a broad attempt to evaluate the country’s health care status [33]. Comparable data on the number and types of functioning health facilities were extracted from the 2012 Annual Report of Iraq’s Ministry of Health. This is the latest report available and is mainly a compilation of institutional and administrative records received from the Directorates of Health [34].

We did not find any discrepancy in the classification of facilities between the two sources that might affected the comparison. Data from both sources appear accurate. The 2003 WHO data were very detailed, including facility name and district code. The 2012 Ministry of Health report did not provide such level of details. To ascertain data quality, we crosschecked information with other reports from previous years and we did not detect any inconsistencies.

The population of each governorate for the year 2003 and 2012 was also obtained from the WHO and Ministry of Health reports. Since no census has been conducted in Iraq after 1997, population data for both years rely on government estimates (see Additional file 1: Table S1) [33,34].

We used these data to quantify progress and setbacks in expanding health service delivery infrastructure. Firstly, we calculated the change in the absolute number of health facilities from early 2003 to the end of 2012. To account for population growth, we computed the change in the number of facilities per 100,000 population. We compared the prevalence of each type of facilities in the autonomous northern Kurdistan region and in the rest of Iraq (centre/south), and among the different governorates. We analysed trends in light of the national plans of reorienting the public health system towards primary health care and attributing a larger role to the private sector for hospital care.

The types of health facilities included in the analysis are: PHCCs (both large and small), public hospitals (all general hospitals at city, district and sub-district levels –if existing– and all specialty hospitals like paediatric, maternity, emergency, surgical, psychiatric and cardiology hospitals), and private hospitals (both secondary and tertiary). Since complete information about types of health services and personnel at each facility and number of beds at each hospital were not available, these important aspects could not be addressed in this paper.

## Results

### Expansion of primary health care centres

Table 1 shows changes in the number of PHCCs between 2003 and 2012. In 2003, there was an average of 5.5 PHCCs per 100,000 population, 2.7 small centres administered by medical auxiliaries and 2.8 large centres administered by doctors. These facilities were unevenly distributed across the country, ranging from 1.9 per 100,000 population in Baghdad to 21.6 in Al-Sulaimaniya. On average, the Kurdistan region exhibited a higher number of PHCCs per 100,000 population than the rest of Iraq.

**Table 1:** **Number of primary health care centres** (**PHCCs**) **in Iraq according to governorates in 2003 and 2012**

**Governorates**	**Small PHCCs per 100,000 population (number)**		**Large PHCCs per 100,000 population (number)**		**Total PHCCs per 100,000 population (number)**		**Change in PPHCs per 100,000 population (number)**
	**2003**	**2012**	**2003**	**2012**	**2003**	**2012**	**2003-2012**
Baghdad	0.1 (5)	0.1 (9)	1.8 (119)	2.9 (207)	1.9 (124)	3.0 (216)	+1.1 (+92)
Basrah	0.4 (8)	0.3 (8)	3.1 (62)	4.3 (113)	3.5 (70)	4.6 (121)	+1.1 (+51)
Nineveh	1.7 (44)	1.6 (54)	2.9 (73)	3.0 (102)	4.6 (117)	4.6 (156)	+0.0 (+39)
Maysan	1.3 (11)	5.1 (51)	2.1 (18)	2.9 (29)	3.4 (29)	8.0 (80)	+4.6 (+51)
Al-Dewaniya	2.3 (21)	2.8 (33)	2.7 (25)	3.3 (38)	5.0 (46)	6.1 (71)	+1.1 (+25)
Diala	1.9 (24)	2.2 (32)	2.3 (29)	4.3 (64)	4.2 (53)	6.5 (96)	+2.3 (+43)
Al-Anbar	5.3 (67)	5.9 (95)	3.9 (50)	4.1 (66)	9.2 (117)	10.0 (161)	+0.8 (+44)
Babylon	2.6 (36)	3.2 (59)	2.3 (32)	2.8 (52)	4.9 (68)	6.0 (111)	+1.1 (+43)
Kerbala	0.5 (4)	2.1 (23)	2.7 (20)	2.6 (28)	3.2 (24)	4.7 (51)	+1.5 (+27)
Kirkuk	2.6 (23)	4.2 (60)	4.1 (36)	3.8 (54)	6.7 (59)	8.0 (114)	+1.3 (+55)
Wasit	0.6 (6)	1.7 (21)	2.7 (25)	3.4 (42)	3.3 (31)	5.1 (63)	+1.8 (+32)
Thi-Qar	1.9 (29)	3.7 (70)	2.1 (32)	3.6 (68)	4.0 (61)	7.3 (138)	+3.3 (+77)
Al-Muthanna	0.2 (1)	3.5 (26)	3.9 (22)	4.2 (31)	4.1 (23)	7.7 (57)	+3.6 (+34)
Salah Al-Deen	3.4 (33)	3.4 (49)	4.0 (39)	3.4 (49)	7.4 (72)	6.8 (98)	-0.6 (+26)
Al-Najaf	2.1 (20)	2.5 (33)	1.5 (14)	3.3 (43)	3.6 (34)	5.8 (76)	+2.2 (+42)
**Centre/South**	**1.4 (332)**	**2.1 (623)**	**2.6 (596)**	**3.3 (986)**	**4.0 (928)**	**5.4 (1,609)**	**+1.4 (+681)**
Erbil	6.4 (86)	10.9 (180)	4.6 (61)	5.4 (90)	11.0 (147)	16.3 (270)	+5.3 (+123)
Dohouk	3.9 (32)	6.7 (78)	5.9 (48)	6.8 (79)	9.8 (80)	13.5 (157)	+3.7 (+77)
Al-Sulaimaniya	17.7 (284)	20.2 (391)	3.9 (63)	5.7 (111)	21.6 (347)	25.9 (502)	+4.3 (+155)
**Kurdistan**	**10.7 (402)**	**13.7 (649)**	**4.6 (172)**	**5.9 (280)**	**15.3 (574)**	**19.6 (929)**	**+4.3 (+355)**
**Total Iraq**	**2.7 (734)**	**3.7 (1,272)**	**2.8 (768)**	**3.7 (1,266)**	**5.5 (1,502)**	**7.4 (2,538)**	**+1.9 (+1,036)**

After a decade, the absolute number of PHCCs increased in all governorates although not everywhere at the same pace. Improvements in the absolute number of facilities were partially, and in a few cases totally, offset by the high rate of population growth. On average, there were 7.4 PHCCs per 100,000 population in 2012, about half of which were large centres administered by doctors. Although the rate of population growth was approximately the same in Kurdistan and central/southern Iraq, the gap in the number of PHCCs per 100,000 population widened from 2003 to 2012, with an average increase of 4.3 PHCCs per 100,000 population in Kurdistan versus an average increase of only 1.4 PHCCs per 100,000 population in centre/south. Differences across governorates also persisted. In 2012, the number of small PHCCs ranged from 0.1 to 5.9 per 100,000 population in the central/southern governorates and from 6.7 to 20.2 in the Kurdish governorates. The number of large centres ranged from 2.6 to 4.3 in the central/southern governorates and from 5.4 to 6.8 in the Kurdish governorates.

### Expansion of public and private hospitals

Changes in the number of public and private hospitals are reported in Table 2. In 2003, there was an average of 0.7 public hospitals per 100,000 population. Differences across governorates were less pronounced than for PHCCs. The number of public hospitals ranged from 0.4 per 100,000 population in Thi-Qar to 1.8 in Al-Sulaimaniya. On average, the number of public hospitals per 100,000 population was higher in the Kurdistan region than in the rest of Iraq.

**Table 2:** Number of public and private hospitals in Iraq according to governorates in 2003 and 2012

**Governorates**	**Public hospitals per 100,000 population (number)**		**Change in public hospitals per 100,000 population (number)**	**Private hospitals per 100,000 population (number)**		**Change in private hospitals per 100,000 population (number)**
	**2003**	**2012**	**2003-2012**	**2003**	**2012**	**2003-2012**
Baghdad	0.6 (38)	0.6 (46)	+0.0 (+8)	0.6 (41)	0.5 (36)	-0.1 (-5)
Basrah	0.5 (10)	0.5 (13)	+0.0 (+3)	0.2 (4)	0.2 (4)	+0.0 (+0)
Nineveh	0.5 (13)	0.4 (14)	-0.1 (+1)	0.2 (4)	0.1 (3)	-0.1 (-1)
Maysan	0.8 (7)	0.6 (6)	-0.2 (-1)	0.1 (1)	0.0 (0)	-0.1 (-1)
Al-Dewaniya	0.8 (7)	0.5 (6)	-0.3 (-1)	0.2 (2)	0.3 (3)	+0.1 (+1)
Diala	0.6 (8)	0.7 (10)	+0.1 (+2)	0.2 (2)	0.2 (3)	+0.0 (+1)
Al-Anbar	0.9 (11)	0.7 (11)	-0.2 (+0)	0.1 (1)	0.1 (2)	+0.0 (+1)
Babylon	0.6 (8)	0.8 (15)	+0.2 (+7)	0.1 (2)	0.2 (4)	+0.1 (+2)
Kerbala	0.7 (5)	0.5 (5)	-0.2 (+0)	0.0 (0)	0.2 (2)	+0.2 (+2)
Kirkuk	0.7 (6)	0.5 (7)	-0.2 (+1)	0.2 (2)	0.1 (2)	-0.1 (+0)
Wasit	1.0 (9)	0.6 (8)	-0.4 (-1)	0.1 (1)	0.0 (0)	-0.1 (-1)
Thi-Qar	0.4 (6)	0.5 (9)	+0.1 (+3)	0.1 (1)	0.1 (2)	+0.0 (+1)
Al-Muthanna	0.7 (4)	0.5 (4)	-0.2 (+0)	0.0 (0)	0.0 (0)	+0.0 (+0)
Salah Al-Deen	0.7 (7)	0.6 (9)	-0.1 (+2)	0.0 (0)	0.1 (2)	+0.1 (+2)
Al-Najaf	0.6 (6)	0.5 (7)	-0.1 (+1)	0.0 (0)	0.2 (3)	+0.2 (+3)
**Centre/South**	**0.6 (145)**	**0.6 (170)**	**+0.0 (+25)**	**0.3 (61)**	**0.2 (66)**	**-0.1 (+5)**
Erbil	0.9 (12)	1.4 (23)	+0.5 (+11)	0.3 (4)	0.8 (13)	+0.5 (+9)
Dohouk	0.9 (7)	1.1 (13)	+0.2 (+6)	0.1 (1)	0.3 (3)	+0.2 (+2)
Al-Sulaimaniya	1.8 (29)	1.7 (33)	-0.1 (+4)	0.1 (2)	0.7 (14)	+0.6 (+12)
**Kurdistan**	**1.3 (48)**	**1.5 (69)**	**+0.2 (+21)**	**0.2 (7)**	**0.6 (30)**	**+0.4 (+23)**
**Total Iraq**	**0.7 (193)**	**0.7 (239)**	**+0.0 (+46)**	**0.3 (68)**	**0.3 (96)**	**+0.0 (+28)**

In 2012, the countrywide average number of public hospitals per 100,000 population was still 0.7. However, the distribution of hospitals across governorates changed significantly. In most central/southern governorates, the limited improvements in the absolute number of public hospitals were completely offset by population growth. As a result, the average number of public hospitals per 100,000 population in centre/south was 0.6 in 2012 as in 2003. By contrast, the Kurdistan region experienced some progress, with the average number of public hospitals per 100,000 population rising from 1.3 to 1.5. At the governorate level, the number of public hospitals in 2012 ranged from 0.4 to 0.8 per 100,000 population in the central/southern governorates and from 1.1 to 1.7 in the Kurdish governorates.

Private hospitals in 2003 were very few and mostly concentrated in Baghdad, where the number per 100,000 population was 0.6. In the other governorates, the number of private hospitals per 100,000 population ranged from 0.0 in Kerbala, Al-Muthanna, Salah Al-Deen and Al-Najaf to 0.3 in Erbil. At that time, the average number of private hospitals per 100,000 population was relatively similar in Kurdistan and centre/south.

Over the period 2003–2012, the number of private hospitals exhibited diverging trends in Kurdistan and central/southern Iraq. In centre/south, the number of private hospitals per 100,000 population declined from 0.3 to 0.2. Some central/southern governorates, including Baghdad, experienced a reduction even in the absolute number of these hospitals. By contrast, in Kurdistan the number of private hospitals per 100,000 population rose from 0.2 to 0.6.

## Discussion

This study has been the first to analyse the expansion of health facilities in post-2003 Iraq. The analysis has revealed some progress, but also many persistent challenges. Over 1,000 new PHCCs and 46 public hospitals were functioning in 2012 compared with 2003. The relatively larger amount of investments in PHCCs than in public hospitals is consistent with the Ministry of Health plan of reorienting the public health sector towards primary care [7,8]. Still in 2012 there was a countrywide average of only 7.4 PHCCs per 100,000 population compared with over 20 PHCCs per 100,000 population in neighbouring Jordan and Iran [35,36]. Efforts to expand the provision of health services were hindered by the high rate of population growth, averaging 2.6% per annum. Due to population growth, the countrywide average number of public hospitals per 100,000 population in 2012 was still 0.7 as in 2003.

There were significant differences in the extent of improvement within the country. In particular, the gap in the average number of PHCCs and public hospitals per 100,000 population between the autonomous Kurdistan region and the rest of Iraq widened. The relatively better status of health infrastructure in Kurdistan originated in the post-1991 period and especially in the years of the Oil for Food Programme (OFFP) between 1996 and 2003. The OFFP was approved by the UN Security Council after 5 years of strict international sanctions and allowed Iraq to use revenues of oil sales for humanitarian needs [37]. The programme was managed directly by UN agencies in Kurdistan and by the Iraqi government in the rest of the country. During this period, new health facilities, particularly PHCCs, were built in Kurdistan by UNICEF and UN-Habitat [38], whereas government investments in health infrastructure in central/southern Iraq were very limited [2].

After 2003, central/southern Iraq has been affected by widespread insurgent and sectarian violence. Security concerns had dramatic consequences on budget allocation and feasibility of health infrastructure projects. For example, almost 50% of Baghdad governorate budget during the years of the occupation was devoted to security, with the health sector receiving only 1% of governorate funds [39]. Since most existing health facilities in centre/south had fallen into disrepair during the sanctions and had suffered further damages following the 2003 invasion, a substantial proportion of total health expenditure had to be used for repairs and renovations [40]. By contrast, the Kurdistan region has remained relatively safe from 2003 onwards. Since there was no fighting in the region, funds from coalition forces were invested mainly in humanitarian fields, including construction of new health facilities [41]. The more secure and stable situation has also allowed the Kurdistan regional government to achieve a higher health expenditure than the central government of Baghdad [39].

The widening gap in health infrastructure between the Kurdistan region and the rest of Iraq is also related to the expansion of the private sector as 23 new private hospitals were opened in Kurdistan. Since 2007, the Kurdistan regional government has adopted a flexible investment policy which has attracted an increasing number of local and foreign investors in a variety of sectors, including health care [42]. The Ministry of Health of Baghdad has also recognised that the private sector has a potentially important role in improving health service provision [7]. However, insecurity and political instability continue to discourage private investments in central/southern Iraq, and the violence-induced outmigration of doctors has led to the closure of a few private hospitals operating during the pre-2003 period [8].

This study adds to the limited documented knowledge about the expansion of health facilities in countries emerging from conflict. It provides an insight into the adverse effect of continuing insecurity and instability on health system recovery, and confirms the importance of inclusive political settlements in enabling successful reconstruction and development plans. The relevance of this paper goes beyond the specific context of Iraq and it can serve as a case study for similar countries where strengthening health infrastructure is a main challenge. A slow pace of reconstruction process due to an uncertain political context has also been noted in other countries emerging from conflict. In the case of Iraq, the comparison between Kurdistan and centre/south makes this particularly evident. For instance, Liberia, Sierra Leone and South Sudan have also experienced disappointingly slow health system rehabilitation efforts in the first few years after the end of major hostilities, due to a lack of legitimacy or weak leadership of the post-conflict governments [15,43,44]. While these countries have gradually overcome the political uncertainty and consolidated their institutions, the political situation of central/southern Iraq a decade after the US-led invasion has remained insecure and fragmented. In fact, the recent wave of violence have further undermined state legitimacy and led to the complete disintegration of health services in the areas controlled by Islamist rebels [45].

Despite the relatively better performance of the Kurdistan region in the expansion of health infrastructure, poor governance, corruption and resource mismanagement have slowed down the pace of development also in this region [26]. More transparent policy-making process and rigorous budgeting and monitoring systems are needed, at both the central and governorate levels, to accelerate progress in the coming years.

The data used in this study have a number of limitations. As noted in the Methods section, information on health facilities for the years 2003 and 2012 were obtained from two different sources, although we did not find any discrepancy or inconsistencies that might undermine the comparison. These data did not permit to address important issues concerning quality of care and equitable access to services. While we assessed changes in the number of health facilities, we could not take into account changes in the size, personnel and types of services provided in these facilities or their distribution between urban and rural areas and between wealthier and poorer districts. Moreover, we could not evaluate the effect that the rapid expansion of a largely unregulated private sector in the Kurdistan region had in terms of high-quality health care provision, and the risk that privatisation may pose in terms of affordability of care and related health inequities. The expansion of facilities is indeed necessary but not sufficient to ensure the right to health care for all Iraqis. Further research is needed to measure the performance and accessibility of public and private health facilities.

## Conclusions

Continuing insecurity and political instability hamper both public and private investments in health infrastructure in countries emerging from conflict and thus pose major challenges to health system recovery. This is particularly evident in the case of Iraq a the decade after the 2003 US-led invasion. The autonomous Kurdistan region, which has been relatively stable from 2003 onwards, has experienced significant progress in expanding the number of public and private health facilities, although still much should be done to reach the standards of neighbouring countries. The situation in the rest of Iraq is a cause for major concern. The slow pace of improvement in the expansion of health facilities is largely attributable to the dire security situation. Due to persistent and growing insecurity, it is unlikely that significant private investments in the health sector will occur in the short-term. This highlights the need for the new Iraqi government, together with international donors, to urgently scale-up resources and commit to strengthening the network of health facilities in underserved areas. Promoting political inclusiveness, transparency in decision-making and accountability in public financial management should be priorities, at both the central and governorate levels.

## Abbreviations

OFFP: Oil for Food Programme; PHCC: Primary health care centre; WHO: World Health Organisation.

## Competing interests

The authors declare that they have no competing interests.

## Authors’ contributions

VC and NPS conceptualised and designed the study. VC carried out the data analysis. VC and NPS prepared the manuscript. Both authors read and approved the final manuscript.

## Supplementary Material

Additional file 1: Table S1 Estimated population of Iraq according to governorates in 2003 and 2012. Click here for file
