# Supplementary material for: Expansion of health facilities in Iraq a decade after the US-led invasion, 2003–2012
Source: Confl Health. 2014 Sep 11;8:16. doi: 10.1186/1752-1505-8-16 (PMC4163049; doi:10.1186/1752-1505-8-16)
Supplement: Additional file 1: Table S1 — Estimated population of Iraq according to governorates in 2003 and 2012. [file 1752-1505-8-16-S1.docx]

**Additional file 1: Table S1 Estimated population of Iraq according to governorates in 2003 and 2012**

| **Governorates** | **Population (thousand)** | | **Annual growth rate (%)** |
| --- | --- | --- | --- |
|  | **2003** | **2012** | **2003-2012** |
| **Baghdad** | 6,500 | 7,255 | +1.2 |
| **Basrah** | 1,982 | 2,602 | +3.1 |
| **Nineveh** | 2,521 | 3,354 | +3.3 |
| **Maysan** | 848 | 997 | +1.8 |
| **Al-Dewaniya** | 916 | 1,162 | +2.7 |
| **Diala** | 1,271 | 1,478 | +1.6 |
| **Al-Anbar** | 1,271 | 1,599 | +2.6 |
| **Babylon** | 1,409 | 1,864 | +3.2 |
| **Kerbala** | 742 | 1,094 | +4.7 |
| **Kirkuk** | 881 | 1,433 | +6.3 |
| **Wasit** | 939 | 1,241 | +3.2 |
| **Thi-Qar** | 1,539 | 1,883 | +2.2 |
| **Al-Muthanna** | 570 | 736 | +2.9 |
| **Salah Al-Deen** | 976 | 1,441 | +4.8 |
| **Al-Najaf** | 950 | 1,320 | +3.9 |
| **Centre/South** | 23,315 | 29,459 | +2.6 |
| **Erbil** | 1,334 | 1,658 | +2.4 |
| **Dohouk** | 817 | 1,159 | +4.2 |
| **Al-Sulaimaniya** | 1,606 | 1,932 | +2.0 |
| **Kurdistan** | 3,757 | 4,749 | +2.6 |
| **Total Iraq** | 27,072 | 34,208 | +2.6 |
